# Genome-Wide Characterization and Expression Profile of the Jumonji-C Family Genes in *Populus alba* × *Populus glandulosa* Reveal Their Potential Roles in Wood Formation

**DOI:** 10.3390/ijms26125666

**Published:** 2025-06-13

**Authors:** Zhenghao Geng, Rui Liu, Xiaojing Yan

**Affiliations:** State Key Laboratory of Tree Genetics and Breeding, Chinese Academy of Forestry, Beijing 100091, China; gengzhenghao@caf.ac.cn (Z.G.); liurui@caf.ac.cn (R.L.)

**Keywords:** JMJ-C family, expression profile, transcription factor, wood formation, *P. alba* × *P. glandulosa*

## Abstract

The Jumonji C (JMJ-C) domain-containing gene family regulates epigenetic and developmental processes in plants. We identified 55 *JMJ-C* genes in *Populus alba* × *Populus glandulosa* using HMM and BLASTp analyses. Chromosomal mapping revealed an asymmetric distribution with conserved synteny. Phylogenetic reconstruction revealed that *PagJMJ* genes segregate into five evolutionarily conserved subfamilies, exhibiting classification patterns identical to those of *Arabidopsis thaliana* and *Populus trichocarpa*. Synteny analysis indicated a closer relationship with *P. trichocarpa* than with *A. thaliana*. Motif and promoter analyses highlighted subfamily-specific features and diverse cis-elements, particularly light-responsive motifs. Expression profiling revealed tissue-specific patterns, with key genes enriched in roots, vascular tissues, and leaves. Developmental analysis in cambium and xylem identified four expression clusters related to wood formation. Co-expression analysis identified six key *PagJMJ* genes (*PagJMJ6*, *29*, *34*, *39*, *53,* and *55*) strongly associated with wood formation-related transcription factors. ChIP-qPCR analysis revealed that key genes co-expressed with *PagJMJ* genes were marked by H3K4me3 and H3K9me2 modifications. These findings provide insights into the evolutionary and functional roles of *PagJMJ* genes in poplar vascular development and wood formation.

## 1. Introduction

Epigenetics refers to reversible, enzyme-mediated modifications that regulate gene expression without altering the DNA sequence, with these changes often being heritable across generations [1,2,3]. Key epigenetic modifications include DNA methylation, histone methylation/acetylation, nucleosome remodeling, and noncoding RNA-mediated regulation [4,5]. Among these, histone methylation is an important and conserved epigenetic modification that regulates transcription by modulating chromatin states or directly recruiting specific effector proteins to activate or repress gene expression [6,7]. The balance of histone methylation is maintained by the opposing actions of histone methyltransferases (HMTs) and histone demethylases (HDMs), which add or remove methyl groups from specific lysine and arginine residues [8,9]. The Jumonji-C (JMJ-C) family proteins are crucial histone demethylases with broad substrate specificity, which remove methyl marks via Fe(II)- and α-ketoglutarate (α-KG)-dependent hydroxylation reactions [10,11]. JMJ-C family proteins can be divided into four categories based on their catalytic domains and substrate specificity: KDM3, KDM4, KDM5, and KDM6 [12]. These proteins are mainly involved in the demethylation process of histone methylation modifications such as H3K4me3, H3K9me2/3, and H3K27me3 [13]. JMJ-C proteins play critical roles in plant developmental processes, including seed germination and dormancy [14,15,16], root patterning [17,18,19], shoot architecture [20,21,22], leaf morphogenesis and senescence [23,24], floral transition [25,26,27,28]. JMJ-C family proteins also play important regulatory roles in plant responses to biotic and abiotic stresses [29,30,31]. JMJ-C-encoding genes have been identified in multiple plant species, such as *Arabidopsis thaliana* [12], *Zea mays* [32], *Oryza sativa* [29], *Solanum lycopersicum* [33], *Citrus sinensis* [34], *Gossypium hirsutum* [31], and *Cucumis melo L.* [35]. Although studies have shown that members of the plant JMJ-C protein family are widely involved in multiple processes such as growth and development, hormone signaling, and environmental stress response, the *JMJ-C* gene family has not been systematically studied in woody plants.

Wood is a key product of plant secondary growth, playing a crucial role in forestry, biomass energy development, carbon sequestration, and ecosystem balance [36]. Its formation is a complex process involving multiple stages, including cell division, differentiation, secondary wall synthesis, and programmed cell death [37,38]. These stages are intricately regulated by genetic, developmental, and environmental factors [39]. To date, most research on the molecular mechanism of wood formation has focused on transcription factors and hormone regulation [40,41,42]. Only a few studies have highlighted the significant role of epigenetic modifications in regulating key genes involved in this process. For example, during secondary wall synthesis, the lignin monomer synthase genes *PtrCCoAOMT2* and *PtrCCR2* are regulated by histone deacetylation [43]. Additionally, in the development of the poplar vascular cambium, the transcription factor PtrVCS2/WOX4a collaborates with a histone acetyltransferase complex to regulate this process [44]. Most existing research focuses on histone acetylation, while the specific regulatory mechanisms of histone methylation in wood formation remain largely unexplored. Therefore, this study focused on the histone demethylase JMJ-C, systematically analyzing its gene family composition, phylogenetic relationships, and expression patterns in *P. alba* × *P. glandulosa* and further explored the potential functional mechanisms in the wood formation.

## 2. Results

### 2.1. Chromosomal Location and Homologous Gene Analysis of JMJ-C Genes in P. alba × P. glandulosa

**Figure 1:**
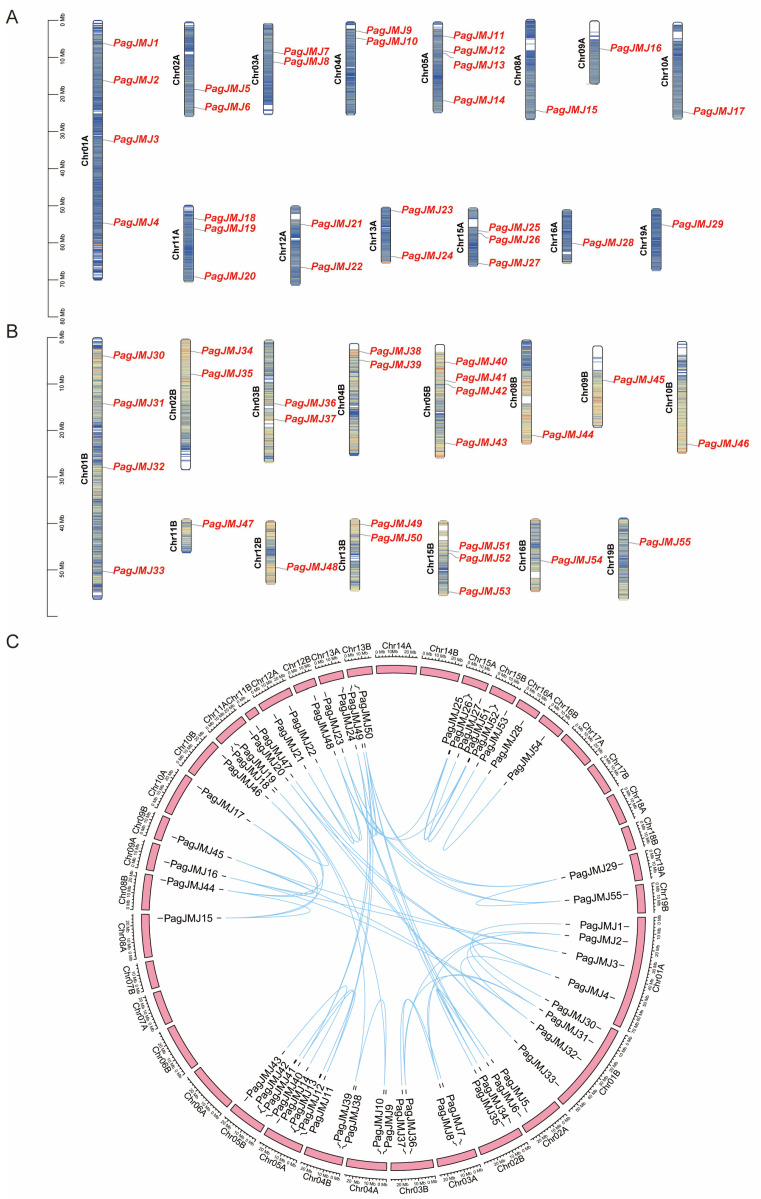
Chromosome distribution and synteny analysis of *PagJMJ* genes in *P. alba* × *P. glandulosa.* (**A**) Chromosomal localization of *PagJMJ* genes across the 12 chromosomes of the A genome in *P. alba* × *P. glandulosa*. (**B**) Chromosomal localization of *PagJMJ* genes across the 12 chromosomes of the B genome in *P. alba* × *P. glandulosa*. (**C**) Comparative analysis of the distribution and syntenic relationships within the *PagJMJ* gene family, with syntenic gene pairs illustrated by blue connecting lines.

Members of the *JMJ-C* gene family were systematically identified in the *P. alba* × *P. glandulosa* genome (v3.1) using a dual approach combining Hidden Markov Models (HMMs) and BLASTp analyses (version 2.16.0). The HMM algorithm was first applied using the conserved JMJ-C domain model (PF02373) as a query, followed by cross-validation against *A. thaliana* JMJ-C protein sequences. This screening identified 55 putative *PagJMJ* genes, designated *PagJMJ1* through *PagJMJ55,* based on their chromosomal positions and linear arrangement. Chromosomal mapping revealed an asymmetric distribution pattern, with genes dispersed across 12 chromosomes in both the A and B chromosome sets (Figure 1A,B). Comparative analysis of homologous chromosome pairs indicated conserved syntenic relationships, with *PagJMJ* genes maintaining corresponding positions in both genome sets. Comprehensive molecular characteristics of PagJMJ proteins—including gene nomenclature, genomic identifiers, chromosomal coordinates, amino acid composition, molecular weight, and theoretical pI values—are detailed in Table S1. The 55 identified proteins exhibited substantial size variation, reflected in diverse molecular weights and isoelectric points. Chromosomal distribution analysis showed no significant correlation between chromosome size and *PagJMJ* gene density.

Homologous gene pairs were systematically identified using MCScanX (Figure 1C), revealing 56 paralogous relationships. Evolutionary pressure analysis showed that all gene pairs had Ka/Ks ratios < 1 (Table S2), indicating predominant purifying selection. Notably, the KDM5 subfamily members *PagJMJ34* and *PagJMJ23* exhibited the highest Ka values, while *PagJMJ34* and *PagJMJ50* had the highest Ks values. The KDM3 subfamily pair—*PagJMJ12* and *PagJMJ41*—displayed the highest Ka/Ks ratios, suggesting relatively relaxed selective constraints within this lineage.

### 2.2. Phylogenetic Analysis of PagJMJ Genes

**Figure 2:**
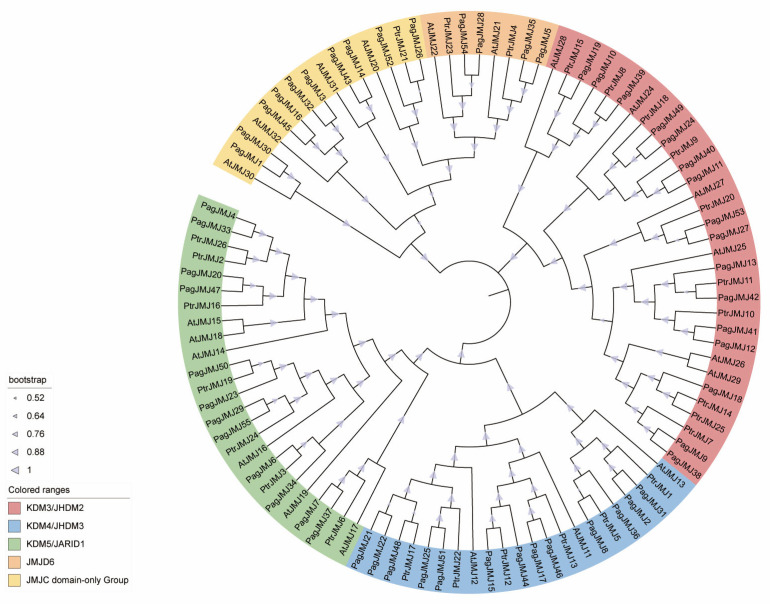
Phylogenetic analysis of JMJ-C family proteins in three plant species. A phylogenetic tree of JMJ-C proteins from *P. alba* × *P. glandulosa*, *P. trichocarpa*, and *Arabidopsis thaliana*. The tree was constructed using the ClustalW and Neighbor-Joining (NJ) method, with 1000 bootstrap replicates in MEGA 12.0. Based on sequence homology, JMJ-C proteins were classified into five distinct subfamilies: KDM4/JHDM3, KDM5/JARID1, JMJD6, KDM3/JHDM2, and JMJ-C domain-only. These subfamilies are represented by different colored blocks, as illustrated in the lower left corner of the figure.

To explore the evolutionary dynamics of *PagJMJ* genes, we conducted a comparative phylogenetic analysis using 102 JMJ-C protein sequences from *A. thaliana* [12], *P. trichocarpa* [45], and *P. alba* × *P. glandulosa* (Figure 2). The phylogenetic tree was divided into five evolutionary clades-KDM4/JHDM3, KDM5/JARID1, JMJD6, KDM3/JHDM2, and JMJ-C domain-only-based on the *Arabidopsis* subfamily classification, supported by sequence conservation patterns and domain architecture. Similar to *Arabidopsis thaliana* and *Populus trichocarpa*, we also identified five subfamilies of JMJ-C proteins in *P. alba* × *P. glandulosa*. Among these clades, KDM4/JHDM3, KDM5/JARID1, and KDM3/JHDM2 formed the three largest groups, while JMJD6 had the fewest members (Figure 2).

### 2.3. Collinearity Analysis of the PagJMJ Genes Family Across Species

**Figure 3:**
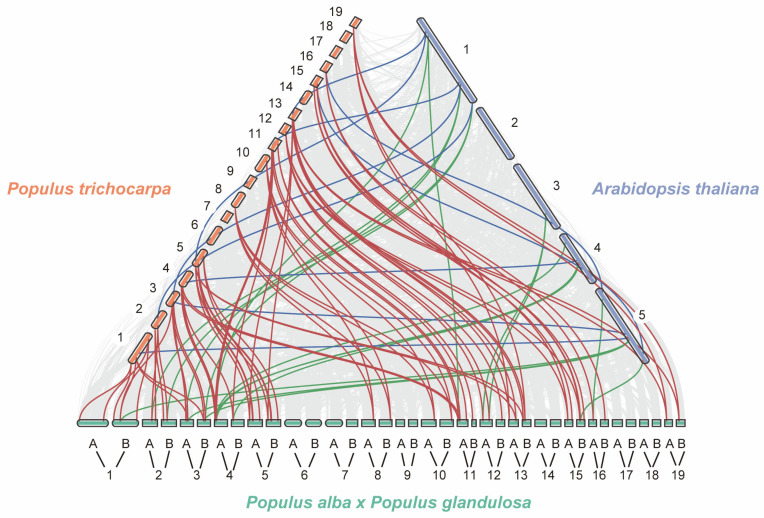
Collinear analysis of *PagJMJ* genes in *P. alba* × *P. glandulosa* with *P. trichocarpa* and *A. thaliana.* Collinear analysis of *PagJMJ* genes from *P. alba* × *P. glandulosa* was performed with two related species, *P. trichocarpa* and *A. thaliana*. Red boxes indicate chromosomes of *P. trichocarpa*, blue boxes indicate chromosomes of *A. thaliana*, green boxes indicate chromosomes of *P. alba* × *P. glandulosa*. Red lines highlight the collinear *JMJ-C* gene pairs between *P. trichocarpa* and *P. alba* × *P. glandulosa*. Blue lines represent the collinear *JMJ-C* gene pairs between *P. trichocarpa* and *A. thaliana*. Green lines denote the collinear *JMJ-C* gene pairs between *P. alba* × *P. glandulosa* and *A. thaliana*.

To explore the evolutionary origins of *PagJMJ* genes, we conducted a synteny analysis to identify interspecies collinearity between *P. alba* × *P. glandulosa* and two other species (Figure 3). Gray lines indicate collinear blocks across different chromosomes, while red, blue, and green lines highlight specific collinear *JMJ-C* gene pairs (red lines: *P. trichocarpa* between *P. alba* × *P. glandulosa*, blue lines: *P. trichocarpa* between *A. thaliana*, green lines: *P. alba* × *P. glandulosa* between *Arabidopsis thaliana*). Evolutionary pressure analysis across these species showed that all gene pairs had Ka/Ks ratios < 1 (Tables S3 and S4), indicating predominant purifying selection. Our analysis identified 13 collinear *JMJ-C* gene pairs between *P. alba* × *P. glandulosa* and *A. thaliana*, with chromosomes 1, 4, and 5 of *A. thaliana* exhibiting collinearity with *PagJMJ* genes. Additionally, 59 collinear *JMJ-C* gene pairs were found between *P. trichocarpa* and *P. alba* × *P. glandulosa*, reflecting their closer evolutionary relationship compared to *A. thaliana*. Notably, several *PagJMJ* genes (*PagJMJ5*, *7*, *8*, *9*, *18*, *28*, *31*, *35*, *36*, *37,* and *52*) showed collinear relationships with eight *JMJ-C* genes in *A. thaliana*, suggesting their potential significance in the evolutionary dynamics of these gene families.

### 2.4. Gene Structure and Conserved Motif Analysis of PagJMJ Genes

**Figure 4:**
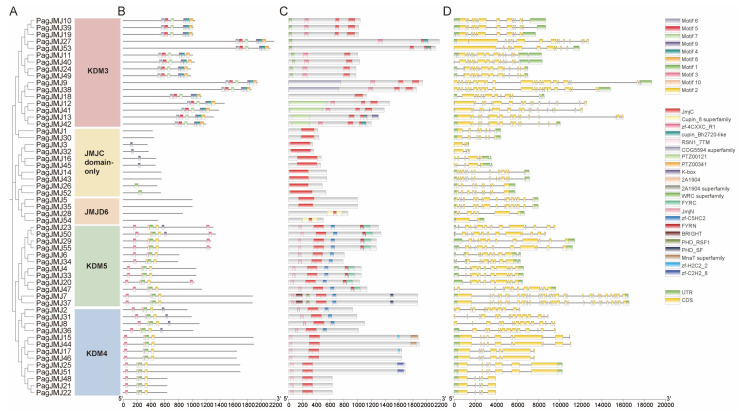
Comprehensive sequence analyses of *PagJMJ* genes. (**A**) Phylogenetic relationships of PagJMJ proteins were reconstructed using the NJ method, revealing that these proteins cluster into three distinct groups. (**B**) Conserved motifs within PagJMJ proteins were identified using the MEME tool (version 5.5.7), resulting in the identification of 10 unique motifs, designated as Motif 1 through Motif 10. (**C**) The schematic representation of conserved domains illustrates their positions and sizes, with each domain depicted as a colored square. (**D**) Gene structure analysis of *PagJMJ* genes highlighted the organization of UTRs, introns, and exons, where UTRs are shown in green, exons in yellow, and introns in grey. A scale bar of 2 kb is provided for reference.

In *P. alba* × *P. glandulosa*, the KDM3/JHDM2, KDM4/JHDM3, KDM5/JARID1, JMJD6, and JMJC domain-only subfamilies consist of 16, 13, 12, 4, and 10 gene members, respectively (Figure 4A). To further characterize the 55 *PagJMJ* genes, we conducted gene structure and motif distribution analyses using the MEME suite (Figure 4B). Ten conserved motifs (1–10) were identified, with the three most conserved motifs overlapping the JMJ-C domain sequence (Figure S1). Subfamily-specific analysis revealed that members of the same group shared similar motif types and arrangements. Notably, motif 9 was present in members of KDM3, KDM4, and KDM5 subfamilies, highlighting the structural conservation of the JMJ-C domain. The KDM3 subfamily, the largest group, consistently retained six motifs (6, 5, 7, 9, 4, and 8). However, variations in motif composition and arrangement within subfamilies suggested potential functional divergence despite shared phylogenetic classification.

Conserved domain analysis confirmed that all *P. alba* × *P. glandulosa* JMJ-C proteins contained the JMJ-C domain (Figure 4C). In the KDM5/JARID1 and KDM4/JHDM3 subfamilies, the JMJ-N domain was consistently present alongside the JMJ-C domain. Additionally, FYRC and FYRN domains were found in PagJMJ23, 50, 29, 55, 4, 33, 20, and 47, while PagJMJ7 and 37 contained the most domains, including JmjN, JmjC, PHD_RSF1, BRIGHT, zf-C5HC2 and PHD_SF (Figure 4C).

To assess functional diversification, we systematically analyzed exon–intron architectures in *PagJMJ* genes (Figure 4D). Structural analysis revealed considerable variation, with exon counts ranging from 2 to 33 and intron numbers varying between 1 and 32. Phylogenetic clustering indicated that genes within the same clade exhibited conserved structural architectures, suggesting shared evolutionary trajectories.

### 2.5. Cis-Element Analysis of PagJMJ Genes Promoter Regions

**Figure 5:**
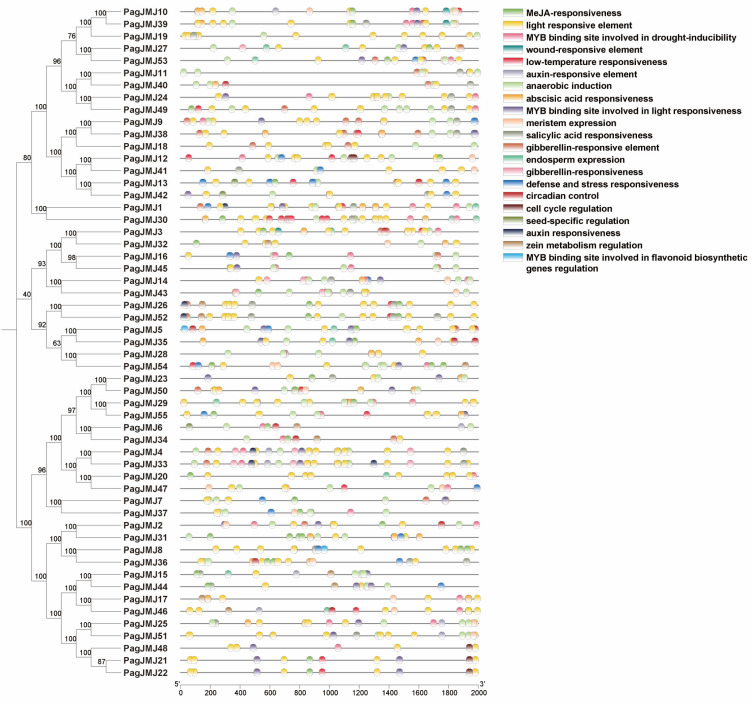
*Cis*-acting elements of the *PagJMJ* genes. The distribution of *cis*-acting elements within the 2000 bp upstream promoter regions of *PagJMJ* genes is illustrated. Different colored circles represent distinct types of regulatory elements, highlighting their positions and abundance across the gene family.

Promoter analysis of *PagJMJ* genes revealed diverse *cis*-acting elements categorized into two functional groups (Figure 5). The first group consists of hormone-responsive elements, including those involved in responses to abscisic acid, methyl jasmonate (MeJA), gibberellin, salicylic acid, and auxin. The second group comprises stress-related elements associated with environmental stimuli such as light, low temperature, drought, and defense responses.

Among these elements, light-responsive motifs were the most abundant, present in all *PagJMJ* family members. Other frequently occurring elements included those associated with anaerobic induction (122 occurrences), abscisic acid responsiveness (68 occurrences), gibberellin responsiveness (68 occurrences), drought inducibility (37 occurrences), and MeJA responsiveness (47 occurrences). Notably, abscisic acid-responsive elements and gibberellin-responsive elements were scarce present in the KDM4 subfamily.

### 2.6. Expression Patterns of PagJMJ Genes in Different Tissues

**Figure 6:**
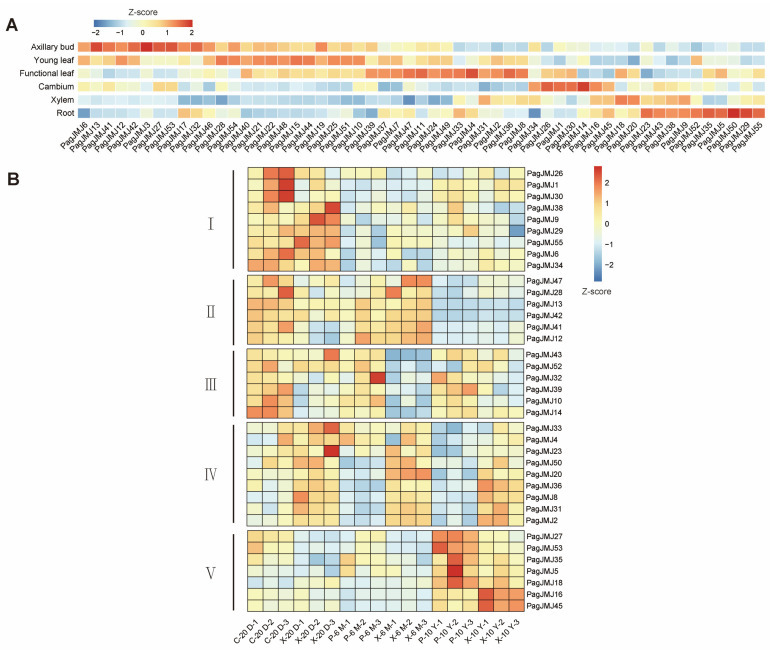
Expression profiles of *PagJMJ* genes in *P. alba* × *P. glandulosa* in 20-day-old cambium (C-20 D) and xylem (X-20 D), 6-month-old phloem (P-6 M) and xylem (X-6 M), and 10-year-old phloem (P-10 Y) and xylem (X-10 Y). (**A**) Heatmap displaying the transcript abundance of *PagJMJ* genes across different tissues, including axillary buds, young leaves, functional leaves, cambium, xylem, and roots. (**B**) Expression patterns of *PagJMJ* genes with high expression levels in cambium and xylem at different developmental stages. K-means clustering analysis categorizes *PagJMJ* genes into five distinct clusters (I–V) based on their expression profiles. The color scale (blue to red) represents low to high expression levels.

To investigate the tissue-specific expression patterns of the *PagJMJ* gene family, we analyzed the *JMJ-C* gene expression levels with reference to the published RNA-seq results of axillary bud, young leaf, functional leaf, cambium, xylem, and root in *P. alba* × *P. glandulosa* [46]. Cluster analysis revealed that these genes formed six distinct expression groups (Figure 6A). These results showed tissue-specific expression profiles across different organs. In root tissues, four genes (*PagJMJ52*, *35*, *5*, *50*, *29,* and *55*) exhibited particularly high expression levels. In the vascular system, *PagJMJ18*, *20,* and *9* were predominantly expressed in the xylem, while *PagJMJ34*, *26*, *1*, *30*, *14*, *16,* and *45* were specifically enriched in the cambium. Leaf tissues displayed diverse expression patterns: twelve genes (*PagJMJ39*, *37*, *7*, *47*, *11*, *24*, *49*, *33*, *4*, *2*, *36,* and *8*) were mainly expressed in functional leaves, whereas another set of ten genes (*PagJMJ28*, *54*, *21*, *22*, *48*, *15*, *44*, *25*, *51,* and *10*) showed high expression in young leaves. Axillary buds exhibited a unique expression profile, with ten genes (*PagJMJ13*, *41*, *42*, *3*, *27*, *53*, *17*, *32*, *46,* and *19*) showing particularly high transcript levels, suggesting their specialized regulatory roles in bud development and maintenance.

To further investigate the relationship between *PagJMJ* genes and wood formation, we selected 37 *PagJMJ* genes with high expression levels in cambium and xylem (key tissues involved in wood development) as targets for follow-up research. Based on the published transcriptome sequencing data from the phloem (including cambium) and xylem of 20-day [46], 6-month [47], and 10-year-old [48] trees, we analyzed the expression patterns of these key genes across different developmental stages. This analysis identified four distinct groups, providing insights into their potential roles in wood formation (Figure 6B). Group I genes (*PagJMJ26*, *1*, *30*, *38*, *9*, *55*, *6,* and *34*) were preferentially expressed in the cambium and xylem of 20-day-old trees. Group II genes (*PagJMJ47*, *28*, *13*, *42*, *41,* and *12*) maintained high expression levels in these vascular tissues at both the 20-day-old and 6-month-old stages. Group III genes (*PagJMJ43*, *52*, *32*, *39*, *10,* and *14*) were consistently expressed in cambium throughout development. Group IV genes (*PagJMJ33*, *4*, *23*, *50*, *20*, *36*, *8*, *31,* and *2*) were consistently expressed in the xylem among different stages. Group V genes (*PagJMJ27*, *53*, *35*, *5*, *18*, *16*, and *45*) were consistently expressed in the vascular tissue at 10-year-old stages. These findings suggest that *PagJMJ* genes may play distinct roles in tissue differentiation and vascular development in poplar.

### 2.7. Co-Expression Analysis of PagJMJ Genes and Wood Formation-Related Genes

Gene co-expression analysis is a powerful approach for identifying functionally related genes with similar expression patterns, revealing potential coregulatory relationships, shared biological functions, or participation in common signaling pathways. To explore the involvement of the 37 *PagJMJ* genes in wood formation, we performed Weighted Correlation Network Analysis (WGCNA) using RNA-Seq data from six tissue types (20-day-old cambium and xylem, 6-month-old phloem and xylem, 10-year-old phloem and xylem). This analysis constructed a tissue-specific co-expression network based on differentially expressed genes.

**Figure 7:**
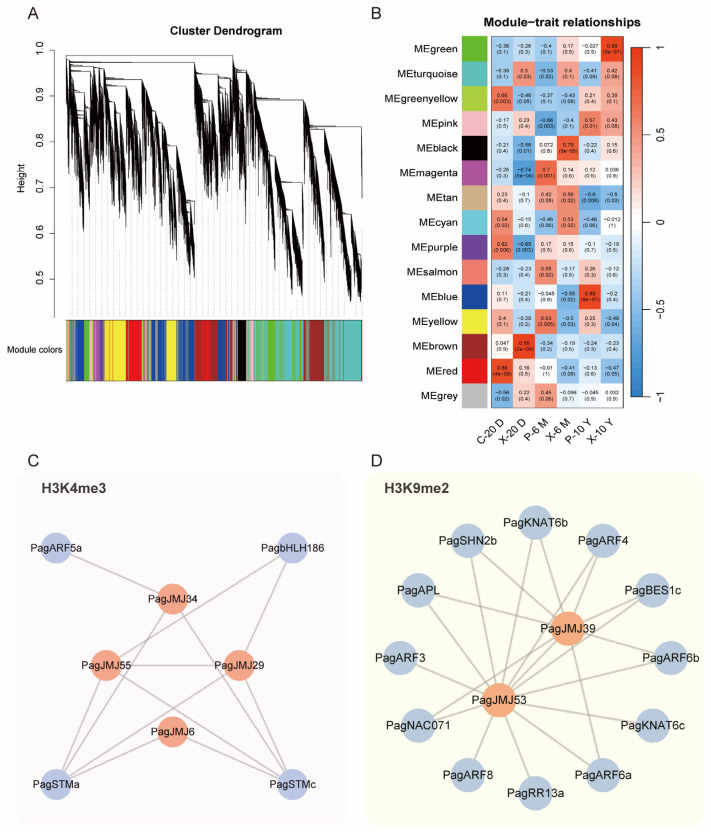
Relationship between *PagJMJ* genes and potential transcription factors predicted by WGCNA. (**A**) Hierarchical cluster tree of gene co-expression modules, with color bands indicating 13 distinct modules. (**B**) Heatmap displaying the correlation between co-expression modules and tissue types. The color intensity represents the correlation coefficient, while values within the cells indicate *p*-values. (**C**,**D**) Co-expression networks of the brown and blue modules, showing interactions between *PagJMJ genes* and potential transcription factors. Red circles represent *PagJMJ* genes, while blue circles indicate transcription factors.

Our results identified 15 distinct co-expression modules (Figure 7A), with strong correlation patterns among genes within each module (Figure 7B). Modules with correlation coefficients greater than 0.7—specifically, the red, brown, magenta, black, blue, and green modules—show significant associations with tissues at different developmental stages. Within the brown and blue modules, we identified eight and two *PagJMJ* genes, respectively. After further selection, we retained four *PagJMJ* genes highly expressed in the xylem and cambium: *PagJMJ6*, *29*, *34,* and *55* in the brown module (Figure 7C) and *PagJMJ39* and *53* in the blue module (Figure 7D). To further investigate the roles of *PagJMJ* genes in wood formation, we identified four and twelve wood formation-related transcription factors (TFs) in the brown and blue modules, respectively. Using a weight threshold of 0.2 for potential interactions, we visualized the regulatory relationships using Cytoscape (3.8.2). The brown module contained key TFs, including *PagbHLH186*, *ARF5a*, and *STMa/c*, while the blue module harbored *PagARF3/4/6a/6b/8*, *KNAT6b/c*, *RR13a*, *SHN2b*, *BES1c*, *NAC071* and *APL* (Figure 7C,D).

**Figure 8:**
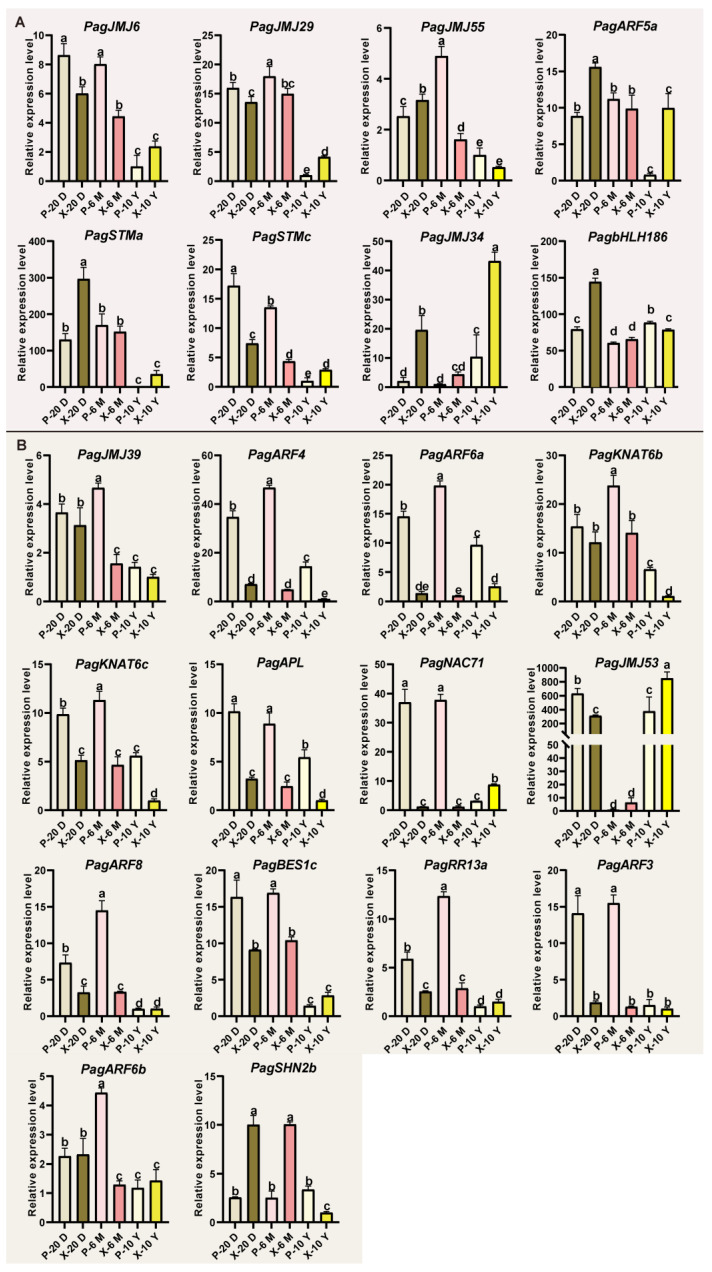
RT-qPCR analysis of *PagJMJ* genes and WGCNA-predicted transcription factors in xylem and phloem tissues of *P. alba* × *P. glandulosa* at different developmental stages. (**A**) Relative expression levels of genes from the brown module were determined by RT-qPCR. (**B**) Relative expression levels of genes from the blue module were determined by RT-qPCR.Expression was analyzed in phloem and xylem tissues collected from 20-day-old (P-20 D, X-20 D), 6-month-old (P-6 M, X-6 M), and 10-year-old (P-10 Y, X-10 Y) trees. Data are presented as means ± standard deviation (SD) of three biological replicates. Statistical significance was determined by one-way analysis of variance (ANOVA), and different lowercase letters indicate significant differences among samples (*p* < 0.05).

We further validated the co-expression relationship of the *PagJMJ* genes and their predicted coregulatory transcription factors in xylem and phloem tissues at three developmental stages: 20 days, 6 months, and 10 years. RT-qPCR analysis revealed that *PagJMJ6*, *JMJ29*, *JMJ55*, *ARF5a*, *STMa,* and *STMc* were highly expressed in both xylem and phloem tissues of younger trees (20-day-old and 6-month-old), suggesting their potential roles in the early stages of wood formation. *PagJMJ34* exhibited elevated expression in the xylem of both 20-day-old and 10-year-old trees, while *PagbHLH186* showed peak expression specifically in the xylem of 20-day-old trees (Figure 8A). In contrast, *PagJMJ39*, *ARF4*, *ARF6a*, *KNAT6b*, *KNAT6c,* and *APL* consistently showed higher transcript levels in phloem compared to xylem across all developmental stages (20 days, 6 months, and 10 years). *PagNAC71* displayed a similar expression pattern to these genes in younger trees, but in 10-year-old trees, its expression was higher in the xylem than in the phloem. *PagJMJ53* mirrored this expression trend with *PagNAC71* in the 20-day-old and 10-year-old trees but showed lower expression in 6-month-old trees. Similarly, *PagARF8*, *BES1c*, *RR13a,* and *ARF3* were more strongly expressed in phloem than in xylem at 20 days and 6 months, although their expression levels did not differ significantly between tissues in 10-year-old trees. *PagARF6b* displayed a phloem-preferential expression pattern only at the 6-month stage, with no significant difference observed at 20 days or 10 years. *PagSHN2b* showed the opposite trend, with higher expression in the xylem at 20 days and 6 months but elevated expression in the phloem in 10-year-old trees (Figure 8B). These findings suggest the following: *PagJMJ6*, *29*, *34,* and *55* may functionally interact with *PagbHLH186*, *ARF5a*, and *STMa/c*; *PagJMJ39 and 53* may functionally interact with *PagARF3/4/6a/6b/8*, *KNAT6b/c*, *APL*, *NAC71*, *BES1c*, *RR13a*, and *SHN2b*. These interactions potentially form regulatory complexes to modulate downstream genes involved in wood formation.

### 2.8. Analysis of Histone Modification Levels in Wood Formation-Related Genes

**Figure 9:**
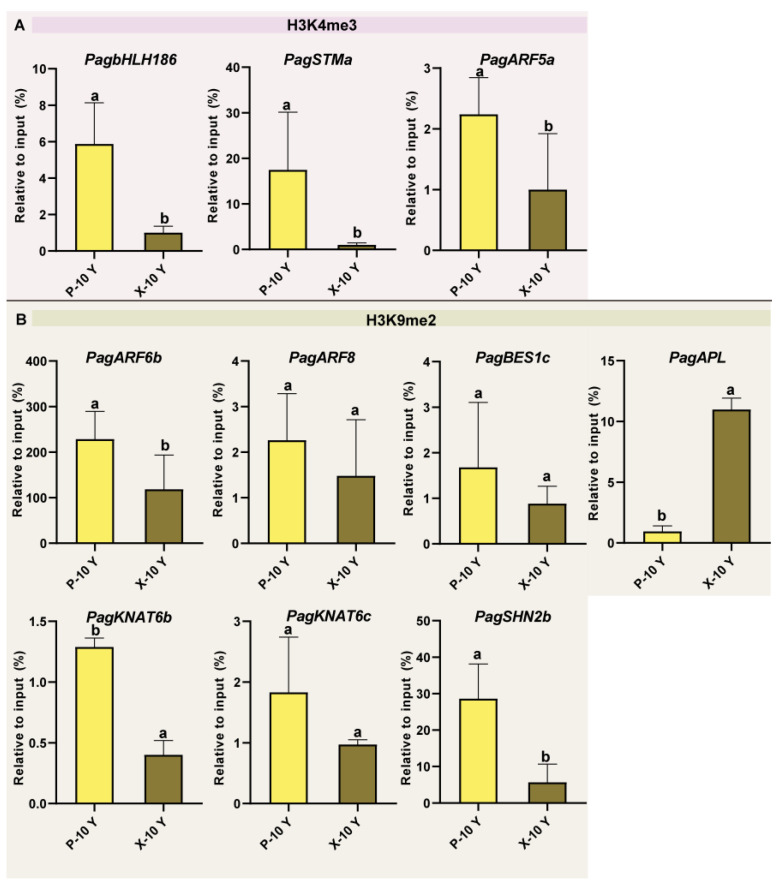
Detection of H3K4me3 (**A**) and H3K9me2 (**B**) levels at specific genes. Phloem and xylem tissues collected from 10-year-old (P-10 Y, X-10 Y) trees were used for the ChIP-qPCR assays with anti-H3K4me3 and anti-H3K9me3 antibodies. Data are presented as means ± standard deviation (SD) of three biological replicates. Statistical significance was determined by one-way analysis of variance (ANOVA), and different lowercase letters indicate significant differences among samples (*p* < 0.05).

Evolutionary analysis revealed that *PagJMJ6/29/34/55* are homologous to *AtJMJ16*, which specifically regulates the demethylation of H3K4me3, a histone modification associated with active transcription [49]. Meanwhile, *PagJMJ39* is homologous to *AtJMJ28,* and *PagJMJ53* shares homology with *AtJMJ27*, while *AtJMJ27* and *AtJMJ28* are responsible for H3K9me2 demethylation, which is typically linked to gene silencing [50]. To further elucidate the functional roles of *PagJMJ* genes, we analyzed H3K4me3 and H3K9me2 modification levels of wood-formation-related genes co-expressed with them. ChIP-qPCR analysis in the phloem and xylem of 10-year-old trees revealed that the *PagbHLH186*, *STMa*, and *ARF5a*, which are co-expressed with *PagJMJ6/29/34/55*, were marked by H3K4me3 modifications, with higher enrichment observed in the phloem compared to the xylem (Figure 9A). Similarly, the *PagARF6b*, *ARF8*, *BES1c*, *APL*, *KNAT6b*, *KNAT6c*, and *SHN2*, which are co-expressed with *PagJMJ39/53*, were enriched for H3K9me2 modifications. Except for *PagAPL*, all of these genes showed higher levels of H3K9me2 in the phloem than in the xylem (Figure 9B).

## 3. Discussion

We identified a total of 55 JMJ-C proteins in *P. alba* × *P. glandulosa*, a number that exceeds those reported in other plant species. All 55 *PagJMJ* genes contained intact coding sequences without premature stop codons, indicating that they encode functional proteins. Specifically, the JMJ-C protein family comprises 21 members in the model *A. thaliana* [12], 22 in *Phyllostachys edulis* [51], 20 in *Oryza sativa* [29], 20 in *Solanum lycopersicum* [33], and 17 in *Cucumis melo L.* [35]. This expansion in *P. alba* × *P. glandulosa* is likely due to its hybrid origin, which results in two sets of chromosomes, as well as an increased number of gene duplication events during evolution. It has been reported in the literature that in species such as *A. thaliana* [12], rice [29], tomato [33], and lemon [35], JMJ-C genes can be divided into five subfamilies. However, there is also a report indicating that in maize [32], the JMJD6 and JMJ-C domain-only subfamilies are lacking. Based on sequence similarity and domain composition, the *PagJMJ* gene family was classified into five subfamilies: KDM4/JHDM3, KDM5/JARID1, KDM3/JHDM2, JMJD6 and JMJ-C domain-only, exhibiting classification patterns identical to those of *A. thaliana* and *P. trichocarpa*. Our findings notably revealed an overlap between the JMJ-C domain and Cupin_8 superfamily in both JMJ-C domain-only and JMJD6 subfamilies (Figure 4C), which aligns with previous reports that JMJ-C proteins belong to the Cupin superfamily due to their characteristic double-stranded β-helix motif structure [52].

Several *PagJMJ* genes exhibited syntenic relationships, suggesting that the expansion of this gene family was primarily driven by segmental duplication rather than tandem duplication. Collinearity analyses further revealed that *P. alba* × *P. glandulosa* shares a higher degree of genomic collinearity with *P. trichocarpa* than with Arabidopsis, consistent with the closer evolutionary relationship among Populus species. The distribution of *PagJMJ* family genes across the two chromosomal sets (A and B) was generally balanced. However, some notable exceptions were observed: chromosomes 11A and 12A each habored three and two *PagJMJ* genes, respectively, whereas their homologous counterparts (11B and 12B) contained only one gene each. These findings suggest potential structural or functional differences between homologous chromosomes that may have influenced the retention or loss of specific *JMJ-C* genes during evolution. Interestingly, light-responsive *cis*-elements were the most abundant among the identified promoter motifs (Figure 5). Although the direct role of light in wood formation remains underexplored in trees, emerging evidence suggests that light signaling pathways can influence vascular differentiation and cambial activity, often through crosstalk with phytohormones such as auxin and cytokinin. Therefore, the enrichment of these elements may indicate a potential mechanism by which *PagJMJ* genes integrate environmental light into the regulation of secondary growth.

Tissue-specific expression analysis provides valuable insights into the functional diversification of *PagJMJ* gene families. Notably, 37 *PagJMJ* genes exhibited preferential expression in the cambium and xylem tissues, suggesting their potential involvement in wood formation. The classification of 37 *PagJMJ* genes into five distinct temporal expression groups suggests a dynamic regulatory network governing vascular tissue differentiation and secondary growth. Histone methylation modification often interacts with transcription factors to exert synergistic regulatory functions and plays an important role in the growth and development of plants. Gene co-expression and qPCR analysis revealed that *PagJMJ* genes (*PagJMJ6*, *29*, *34*, *39*, *53,* and *55*) exhibit strong associations with key transcription factors involved in vascular development, secondary growth, and hormone signaling pathways.

JMJ proteins, as histone demethylases, play essential roles in epigenetic regulation by modulating histone methylation status. ChIP-qPCR results in phloem and xylem tissues showed that *PagbHLH186*, *STMa*, and *ARF5a* are associated with H3K4me3 enrichment, whereas *PagARF6b*, *ARF8*, *BES1c*, *APL*, *KNAT6b*, *KNAT6c*, and *SHN2* is enriched for H3K9me2. These patterns are consistent with the histone modification targets of their respective co-expression *PagJMJ* genes, supporting their potential regulatory roles in wood development. Given these co-expression and ChIP-qPCR analysis results, we speculate that PagJMJ6, PagJMJ29, PagJMJ34, and PagJMJ55 may regulate wood formation by modifying the H3K4me3 methylation status of key gene loci. Meanwhile, PagJMJ39 and PagJMJ53 may influence wood formation through H3K9me2 modification, potentially altering chromatin accessibility and gene expression patterns. Further experimental validation, such as protein-DNA interaction profiling and genetic modification studies, will be necessary to elucidate their precise regulatory roles and molecular mechanisms in secondary growth and wood development.

## 4. Materials and Methods

### 4.1. Plant Materials

Poplar (*P. alba* × *P. glandulosa*) plants were planted in the greenhouse located at the State Key Laboratory of Tree Genetics and Breeding, Beijing, China. The xylem and phloem of 20-day-old and 6-month-old plants were extracted and stored in liquid nitrogen for RNA extraction. The xylem and phloem samples of 10-year-old plants were collected from *P. alba* × *P. glandulosa* trees in the Beiwu garden (39°59′ N, 116°15′ E; Beijing, China).

### 4.2. Identification and Physicochemical Analysis of PagJMJ Proteins

Genomic resources for this study were obtained from publicly available databases. Genome sequences and annotation files for *P. trichocarpa* (v3.1) and *A. thaliana* (TAIR10) were retrieved from the Phytozome database (https://phytozome-next.jgi.doe.gov/ (accessed on 3 March 2025)), while genome data for *P. alba* × *P. glandulosa* (v3.1) were acquired from Figshare (https://doi.org/10.6084/m9.figshare.12369209 (accessed on 3 March 2025)). To identify JMJ-C proteins, we employed a dual-approach strategy. First, BLASTp searches were conducted using Arabidopsis JMJ-C protein sequences as queries against both Populus genomes, applying a stringent E-value cutoff of 1 × 10^−10^. Second, the conserved JMJ-C domain HMM profile (PF02373) was downloaded from the InterPro database (https://www.ebi.ac.uk/interpro/ (accessed on 3 March 2025)) and used for HMMER 3.4 searches with an E-value threshold of 1 × 10^−5^. The results from both methods were cross-validated and integrated with existing literature to ensure comprehensive identification without redundancy. For the 55 genes obtained above, we resubmitted their protein sequences to the InterPro database for verification and found that each gene contained the JMJ-C domain.

For phylogenetic analysis, JMJ-C protein sequences were aligned and trimmed using ClustalW, and a phylogenetic tree was conducted using MEGA12 with the Neighbor-Joining method. The analysis included 26 JMJ-C proteins from *P. trichocarpa*, 55 from *P. alba* × *P. glandulosa*, and 21 from *A. thaliana*, employing the p-distance model with 1000 bootstrap replicates. The phylogenetic tree was visualized using iTOL (https://itol.embl.de/ (accessed on 5 March 2025)). Additionally, physicochemical properties of the identified PagJMJ proteins, including molecular weight and isoelectric point, were characterized using the ExPASy ProtParam tool (http://web.expasy.org/protparam/ (accessed on 5 March 2025)).

### 4.3. Chromosomal Localization and Collinearity Analysis of PagJMJ Genes

The chromosomal positions of *PagJMJ* genes in the *P. alba* × *P. glandulosa* genome were determined using GFF annotations and visualized with the Gene Location Visualize module in TBtools (version 2.210) [53]. Using MCScanX (version 2019), collinearity analysis was performed to identify conserved syntenic relationships among *PagJMJ* genes. The results were visualized using the *circlize* and *ComplexHeatmap* packages in R. We calculated the ratio of non-synonymous to synonymous substitution (Ka/Ks) for duplicated gene pairs using the YN method in KaKs_Calculator (version 3.0) to assess evolutionary constraints [54].

### 4.4. Analysis of Conserved Domains, Motifs, Gene Structures and Cis-Acting Elements

Conserved domains and motifs in PagJMJ proteins were identified using MEME (http://meme-suite.org/tools/meme (accessed on 10 March 2025)). Gene structures, including intron–exon organization and untranslated regions (UTRs), were annotated based on GFF3 data from the *P. alba* × *P. glandulosa* genome database. For *cis*-acting regulatory element analysis, 2000 bp upstream promoter sequences of all *PagJMJ* genes were extracted and analyzed using the PlantCARE database (http://bioinformatics.psb.ugent.be/webtools/plantcare/html/ (accessed on 10 March 2025)) [55]. All results were visualized using TBtools (version 2.210) [53].

### 4.5. Collinearity Analyses of JMJ-C Genes Across Different Species

The protein sequences files and GFF annotations of *P. trichocarpa*, *P. alba* × *P. glandulosa*, and *A. thaliana* were aligned and analyzed using the jcvi.compara.catalog module of JCVI software (v1.4.16, Tanghaibao; Fuzhou, China). Collinearity relationships were then visualized using the jcvi.graphics.karyotype module.

### 4.6. Transcriptomic Resources

To investigate the expression patterns of *PagJMJ* genes, we utilized transcriptomic data from different tissues (axillary bud, young leaf, functional leaf, cambium, xylem, and root), available in the NCBI Sequence Read Archive (SRA) under accession number PRJNA916663. Additionally, RNA-seq data from different development stages (6-month-old and 10-year-old) were retrieved from the SRA with accession numbers PRJNA723483 and PRJNA703312, respectively. Gene expression levels were visualized in heatmaps, with normalization performed using Z-score transformation.

### 4.7. Weighted Gene Co-Expression Network Analysis (WGCNA)

Gene co-expression networks were constructed and analyzed using the WGCNA package in R. Only genes with expression levels >1 in at least 90% of samples were included. The weighted gene co-expression network was constructed with the following parameters: a soft-thresholding power (β) of 11 to enforce scale-free topology, a signed hybrid network type, module detection sensitivity set to DeepSplit = 4, a minimum module size of 30 genes, and module merging at a cut height of 0.35. Then, we exported the network files for Cytoscape visualization using WGCNA’s exportNetworkToCytoscape function with an edge weight threshold of 0.2. The resulting networks, including *PagJMJ* genes, were visualized using Cytoscape (v3.8.2).

### 4.8. RNA Extraction and Quantitative RT-PCR

Total RNA was extracted from the xylem and phloem tissues of *P. alba* × *P. glandulosa* at three developmental stages (20-day-old, 6-month-old, and 10-year-old) using the CTAB method. cDNA was synthesized from 1 µg of total RNA using the HiFiScript gDNA Removal RT MasterMix (CWBIO, CW2020M, Nanjing, China). RT-qPCR was performed using the UltraSYBR Mixture (CWBIO, Nanjing, China) with 50× diluted cDNA as the template. The 18S rRNA gene served as the internal reference.

The RT-qPCR conditions consisted of an initial denaturation at 95 °C for 10 min, followed by 45 cycles of 95 °C for 15 s and 60 °C for 1 min. Relative gene expression levels were calculated using the 2^−ΔΔCt^ method. Three independent biological replicates and three technical replicates were analyzed for each sample. Data were expressed as mean ± standard deviation (SD) and visualized using GraphPad Prism 8. Gene-specific primers were listed in Table S5.

### 4.9. Chromatin Immunoprecipitation and Quantitative PCR

Chromatin immunoprecipitation (ChIP) assays were performed according to established protocols [56]. Briefly, 5 g of xylem and phloem tissues from 10-year-old poplar trees were ground in liquid nitrogen and suspended in precooled lysis buffer. Crosslinking was carried out by vacuum infiltration with 1% (*w*/*v*) formaldehyde for 20 min and subsequently quenched with 0.125 M glycine. Chromatin was then extracted and sonicated using a Bioruptor Plus (Diagenode, Basel, Switzerland) to generate DNA fragments of 200–500 bp. Immunoprecipitation was performed overnight at 4 °C using antibodies specific to H3K4me3 and H3K9me2. After reverse crosslinking, DNA was purified and subjected to quantitative PCR (qPCR) analysis. Gene-specific primers were listed in Table S5.

## 5. Conclusions

Based on phylogenetic, genomic distribution, gene structure and conserved motif, promoter binding site, and expression profiles analysis, this study comprehensively and systematically identified 55 *PagJMJ* genes in *P. alba* × *P. glandulosa*. Tissue-specific expression and gene co-expression analysis revealed that *PagJMJ* genes (*PagJMJ6*, *29*, *34*, *39*, *53,* and *55*) may play a role in modulating TF expression (*PagbHLH186*, *ARF5a*, *STMa/c*, *ARF3/4/6a/6b/8*, *KNAT6b/c*, *RR13a*, *SHN2b*, *BES1c*, *NAC071,* and *APL*) during wood formation. Overall, these findings highlight the potential regulatory roles of *PagJMJ* genes in wood formation and provide new insights into the epigenetic and transcriptional control of secondary growth in poplar.

## Data Availability

The original contributions presented in this study are included in the article. Further inquiries can be directed to the corresponding author.

## Supplementary Materials

The following supporting information can be downloaded at https://www.mdpi.com/article/10.3390/ijms26125666/s1.
