# Supplementary material for: Genome-Wide Characterization and Expression Profile of the Jumonji-C Family Genes in Populus alba × Populus glandulosa Reveal Their Potential Roles in Wood Formation
Source: Int J Mol Sci. 2025 Jun 13;26(12):5666. doi: 10.3390/ijms26125666 (PMC12193463; doi:10.3390/ijms26125666)
Supplement: Supplementary file 1 [file ijms-26-05666-s001.zip › Supplementary Figure S1.pdf]

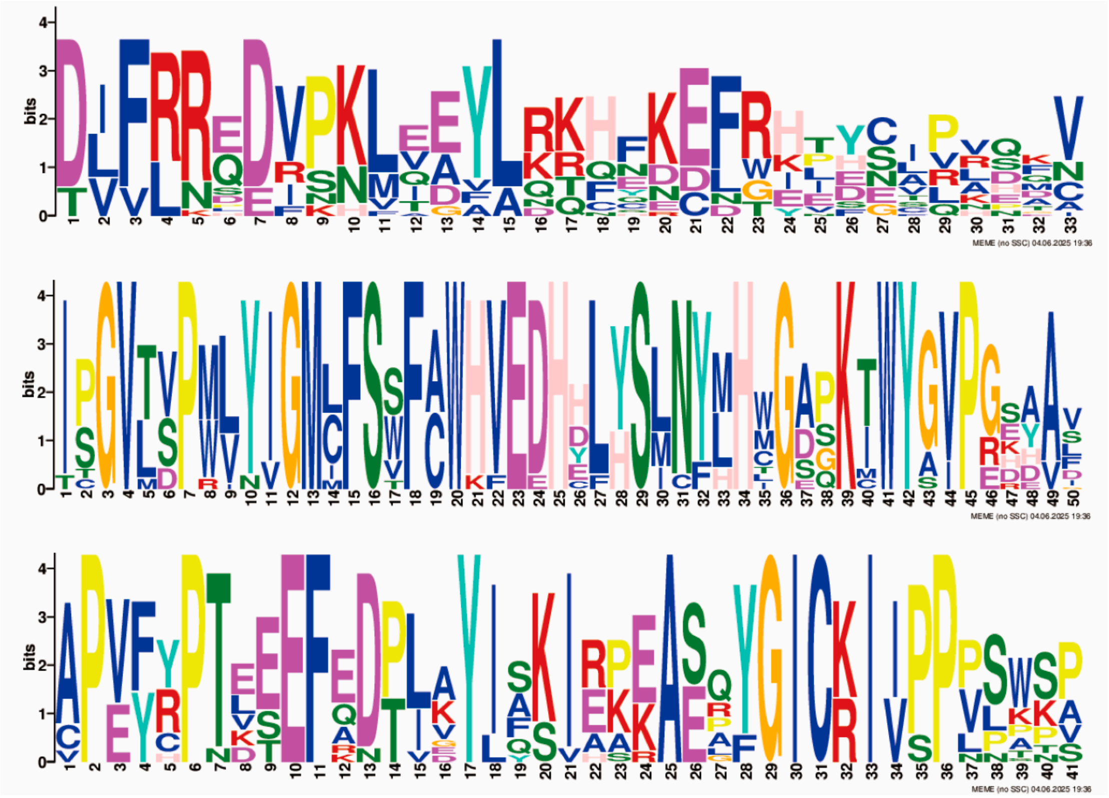

Figure S1 Visualization of multiple sequence alignment of the *PagJMJ* family DNA binding domains.

The sequence logo illustrates the degree of conservation across the DNA-binding domains of PagJMJ proteins. The overall height of each letter stack represents the sequence conservation at that position, measured in bits. Within each stack, the relative height of individual amino acid letters reflects their frequency at that specific position, indicating conserved and variable residues across the family.
